# Supplementary material for: MicroRNA-9-5p-CDX2 Axis: A Useful Prognostic Biomarker for Patients with Stage II/III Colorectal Cancer
Source: Cancers (Basel). 2019 Nov 27;11(12):1891. doi: 10.3390/cancers11121891 (PMC6966658; doi:10.3390/cancers11121891)
Supplement: Supplementary file 1 [file cancers-11-01891-s001.pdf]

Supplementary Materials

MicroRNA-9-5p-CDX2 Axis: A Useful Prognostic Biomarker for Patients with Stage II/III Colorectal Cancer

Aya Nishiuchi [1], Shigeo Hisamori [1,*], Masazumi Sakaguchi [1,2], Keita Fukuyama [3], Nobuaki Hoshino [1], Yoshiro Itatani [1], Shusaku Honma [1], Hisatsugu Maekawa [1], Tatsuto Nishigori [1], Shigeru Tsunoda [1], Kazutaka Obama [1], Hiroyuki Miyoshi [4], Yohei Shimono [5], M. Mark Taketo [6] and Yoshiharu Sakai [1]

Supplementary Figures

Figure S1

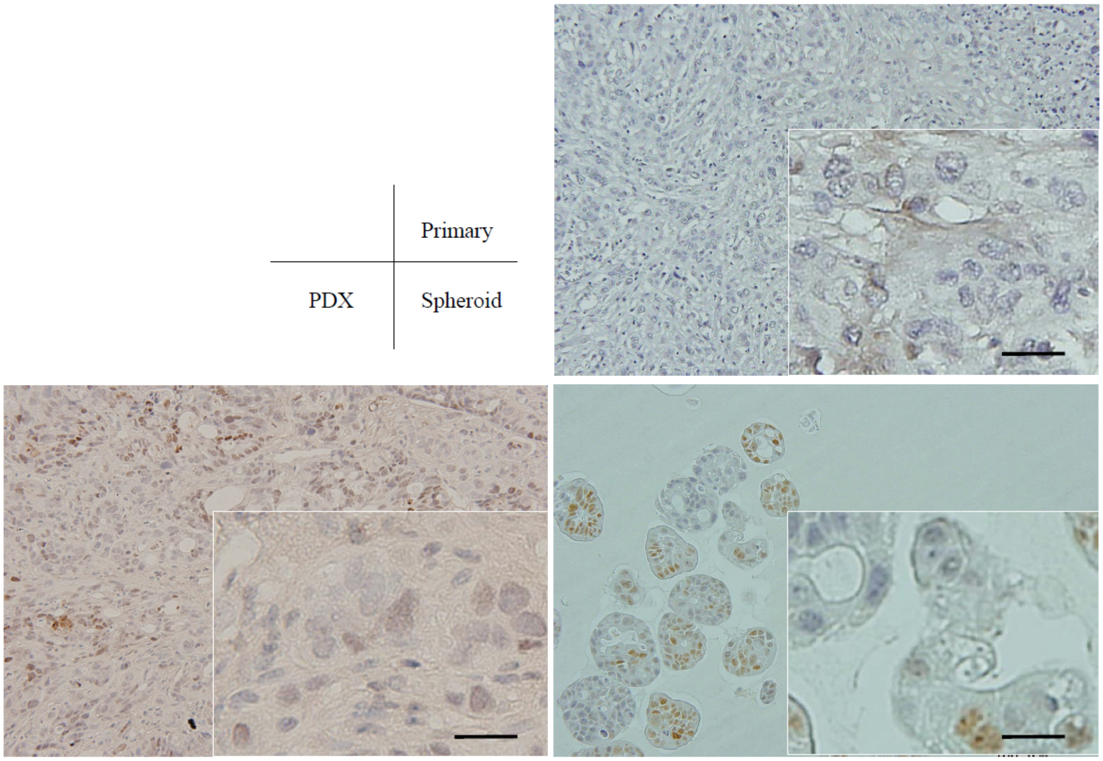

Figure S1. Similar staining pattern of CDX2 in primary tumor, PDX and spheroid by IHC examination. The scale bar represents 100 μm.

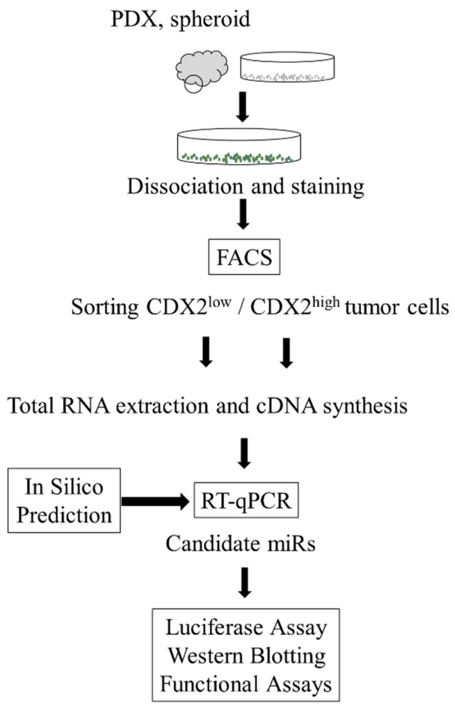

Figure S2. Schematic workflow to identify the miR that regulates the expression of CDX2.
